# Cost-utility analysis of percutaneous mitral valve repair in inoperable patients with functional mitral regurgitation in German settings

**DOI:** 10.1186/s12872-015-0039-8

**Published:** 2015-05-14

**Authors:** Oleg Borisenko, Michael Haude, Uta C Hoppe, Tomasz Siminiak, Janusz Lipiecki, Steve L Goldberg, Nawzer Mehta, Omari V Bouknight, Staffan Bjessmo, David G Reuter

**Affiliations:** 1 Synergus AB, Svardvagen 19, 182 33 Danderyd, Sweden; 2 Medical Clinic I, Neuss City Clinic, Lukas Krankenhaus GmbH, Preußenstraße 84, 41464 Neuss, Germany; 3 Department of Internal Medicine II, Paracelsus Medical University Salzburg, Müllner Hauptstr. 48, A-5020 Salzburg, Austria; 4 Department of Cardiology, Interventional Cardiology, HCP Medical Center, Poznan University of Medical Sciences, ul. 28 czerwca 1956 Nr 194, 61-485 Poznan, Poland; 5 Pole Sante Republique, 105 Avenue de la République, 63050 Clermont-Ferrand, France; 6 Department of Cardiology, University of Washington, Seattle, WA USA; 7 Cardiac Dimensions Incorporated, 5540 Lake Washington Blvd NE, Kirkland, WA 98033 USA; 8 Department of Learning Informatics, Medical Management Centre, Management and Ethics, Karolinska Institutet, Solnavägen 1, 171 77 Solna, Sweden; 9 Division of Pediatric Emergency Medicine, Seattle Children’s Hospital, 4800 Sand Point Way NE, Seattle, WA 98105 USA

**Keywords:** Heart failure, Functional mitral regurgitation, Percutaneous valve repair treatment, Cost-utility analysis, Germany

## Abstract

**Background:**

To determine the cost-effectiveness of the percutaneous mitral valve repair (PMVR) using Carillon® Mitral Contour System® (Cardiac Dimensions Inc., Kirkland, WA, USA) in patients with congestive heart failure accompanied by moderate to severe functional mitral regurgitation (FMR) compared to the prolongation of optimal medical treatment (OMT).

**Methods:**

Cost-utility analysis using a combination of a decision tree and Markov process was performed. The clinical effectiveness was determined based on the results of the Transcatheter Implantation of Carillon Mitral Annuloplasty Device (TITAN) trial. The mean age of the target population was 62 years, 77 % of the patients were males, 64 % of the patients had severe FMR and all patients had New York Heart Association functional class III. The epidemiological, cost and utility data were derived from the literature. The analysis was performed from the German statutory health insurance perspective over 10-year time horizon.

**Results:**

Over 10 years, the total cost was €36,785 in the PMVR arm and €18,944 in the OMT arm. However, PMVR provided additional benefits to patients with an 1.15 incremental quality-adjusted life years (QALY) and an 1.41 incremental life years. The percutaneous procedure was cost-effective in comparison to OMT with an incremental cost-effectiveness ratio of €15,533/QALY. Results were robust in the deterministic sensitivity analysis. In the probabilistic sensitivity analysis with a willingness-to-pay threshold of €35,000/QALY, PMVR had a 84 % probability of being cost-effective.

**Conclusions:**

Percutaneous mitral valve repair may be cost-effective in inoperable patients with FMR due to heart failure.

## Background

Functional mitral regurgitation (FMR) is a common condition that can occur secondary to systolic heart failure and dilated left ventricular cardiomyopathy [1–4]. The cornerstone of clinical management is medical therapy with surgical valve replacement or repair, which is not possible in severely diseased patients with contraindications to surgery and general anesthesia [5, 6]. Although FMR can lead to increased mortality, reduced functional capacity and increased healthcare cost, only a few targeted treatments, addressing FMR, are available. There is ongoing debate about the optimal intervention for FMR, its timing and effectiveness [7–11].

The Carillon® Mitral Contour System® (Cardiac Dimensions Inc., Kirkland, WA, USA) is a novel percutaneous coronary sinus-based mitral annuloplasty device designed to treat FMR. This approach has been shown to significantly reduce FMR, improve functional capacity and quality of life as well as induce reverse left ventricular remodeling [12–14]. Given these findings and the high prevalence of patients with heart failure and FMR, it is important to understand the economic implications of percutaneous mitral valve repair (PMVR). After safety, efficacy and effectiveness aspects, cost-effectiveness is becoming the fourth hurdle for reimbursement of novel technologies in most developed countries [15–17]. The current clinical evidence for CE-marked Carillon® Mitral Contour System® is based on the single arm AMADEUS [12] and the non-randomized controlled Transcatheter Implantation of Carillon Mitral Annuloplasty Device (TITAN) [13] trials, which makes it possible to perform an early economic evaluation to inform decision-making about the cost-effectiveness of PMVR and ensure the timely access of patients to this treatment option.

The objective of the study was to determine, in German settings, the cost-effectiveness of PMVR using the Carillon® Mitral Contour System® in patients with congestive heart failure and moderate to severe FMR with normal QRS interval, compared with the prolongation of optimal medical treatment.

## Methods

### Model description

**Fig. 1:**
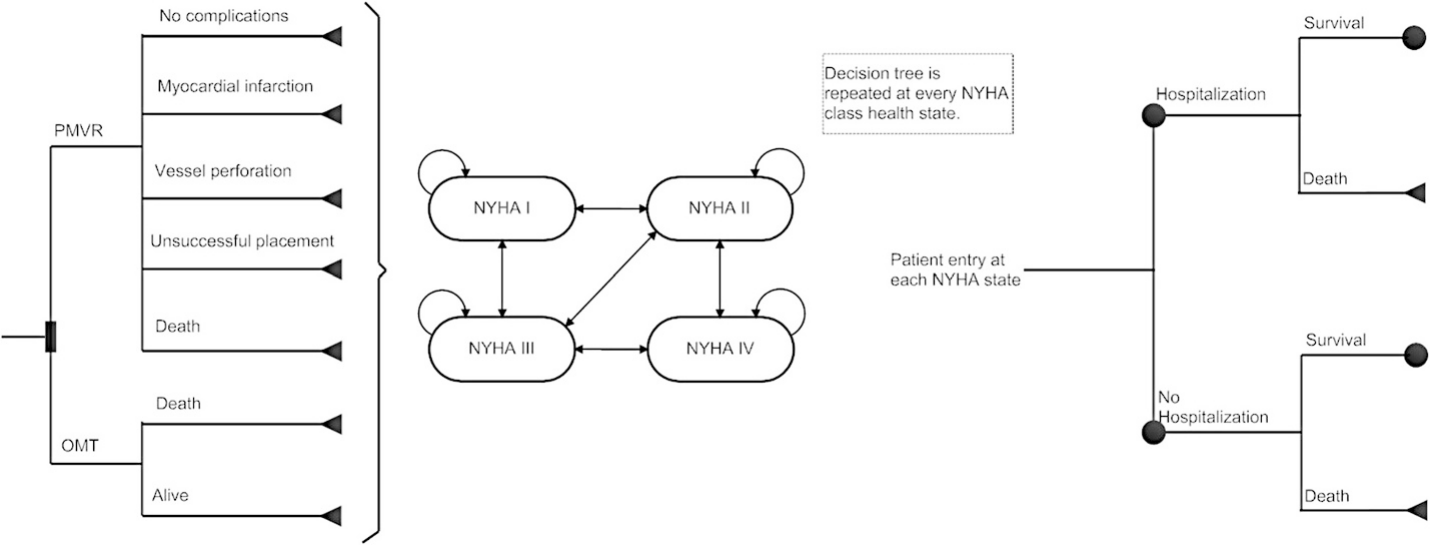
Decision tree and Markov model structure. NYHA, New York Heart Association; OMT, optimal medical treatment; PMVR, percutaneous mitral valve repair

The combination of a decision tree and a Markov process [18–20] was used to assess the economic value of percutaneous annuloplasty in patients with FMR. The cycle length was one month. The model starts with a decision tree (Fig. 1), in which the patients undergoing PMVR may have several possible outcomes, including discharge from the hospital without complications, peri-operative complications with subsequent discharge from the hospital, unsuccessful device implantation with subsequent removal of the device during the initial procedure or death. All serious complications related to the implantation procedure were selected from the published TITAN trial [13]. During the first month of the model, the patients in the optimal medical treatment arm could die or stay alive. Decision tree estimates the cost-effectiveness for the first month in the model. Thereafter, all patients in both arms entered the Markov model.

The structure of the follow-up Markov model was applied to both treatment arms. The Markov model consists of four health states representing the four New York Heart Association (NYHA) functional classes. Patients may progress from one NYHA class to another. In each state, hospitalization and death are possible. The probability of hospitalization and death is higher with increasing NYHA classes. Each state of the Markov model has associated cost and utility based on the status of health-related quality of life.

The basis for the analysis is that PMVR, by improving symptoms (i.e., improving the patient’s NYHA functional class) through the reduction of FMR, will reduce both mortality and hospitalizations compared with the prolongation of optimal medical treatment.

### Clinical inputs

Search for relevant clinical, cost and utility inputs was performed in the Medline database using key words “functional mitral regurgitation”, “prognosis”, “mortality”, “cost”, “NYHA class” in April 2012 and repeated in August 2014.

Monthly transition probabilities across NYHA classes for the Carillon and optimal medical therapy arms (Table 1) were derived from the TITAN trial [13]. The TITAN trial enrolled fifty-three patients with dilated ischemic or non-ischemic cardiomyopathy from seven European centers. The inclusion criteria were 1) at least moderate (2+) FMR, 2) a left ventricular ejection fraction (LVEF) <40 %, 3) NYHA class II-IV, 4) a 6 min walk distance between 150 and 450 m and 5) a stable heart failure medication regimen. Thirty-six patients underwent permanent device implantation and seventeen patients had the device recaptured intra-operatively (i.e., device was removed) either for clinical indications (i.e., transient coronary compromise) or for a protocol defined indication (i.e., <1 grade FMR reduction). Both groups underwent serial follow-ups with the non-implanted cohort serving as a non-randomized, non-blinded comparator group. Data for 17 patients with device recaptured after unsuccessful implantation have been used to obtain transition probabilities in the optimal medical management (control) arm in the model. Compared with the control group, clinically and statistically significant reduction in FMR (regurgitant volume, effective regurgitant orifice area (EROA), vena contracta, mitral regurgitation jet area/left atrial area) was demonstrated. Different transition probabilities were applied for the first month, for months 2 to 11, and from month 12 and onwards. As the TITAN trial provided limited data about the transitions from NYHA class IV to other classes, an assumption was made about these transitions being similar to transitions from NYHA class III. It was assumed that NYHA class I does not lead to increased mortality. Clinical inputs are presented in Table 2.

**Table 1:** Transition probabilities between NYHA functional classes [13]

Transition probabilities in cycle 1				
	NYHA I	NYHA II	NYHA III	NYHA IV
Optimal medical treatment arm				
NYHA Class III	0.00	0.17	0.75	0.08
PMVR arm				
NYHA Class III	0.10	0.73	0.13	0.03
Transition probabilities in cycles 2–11				
	NYHA I	NYHA II	NYHA III	NYHA IV
Optimal medical treatment arm				
NYHA Class I	0.00	0.00	0.00	0.00
NYHA Class II	0.00	0.67	0.00	0.33
NYHA Class III	0.00	0.17	0.83	0.00
NYHA Class IV	0.00	0.00	0.17	0.83
PMVR arm				
NYHA Class I	0.25	0.75	0.00	0.00
NYHA Class II	0.11	0.63	0.26	0.00
NYHA Class III	0.00	0.50	0.50	0.00
NYHA Class IV	0.00	0.00	0.50	0.50
Transition probabilities in cycle 12 and onwards				
	NYHA I	NYHA II	NYHA III	NYHA IV
Optimal medical treatment arm				
NYHA Class I	0.00	0.00	0.00	0.00
NYHA Class II	0.00	0.50	0.50	0.00
NYHA Class III	0.00	0.67	0.33	0.00
NYHA Class IV	0.00	0.00	0.67	0.33
PMVR arm				
NYHA Class I	0.67	0.33	0.00	0.00
NYHA Class II	0.12	0.71	0.18	0.00
NYHA Class III	0.00	0.40	0.60	0.00
NYHA Class IV	0.00	0.00	0.40	0.60

NYHA, New York Heart Association; PMVR, percutaneous mitral valve repair

**Table 2:** Clinical, cost and utility inputs

Variable	Base Case Value	Range	Distribution for probabilistic sensitivity analysis	Reference
Characteristics of patients’ population				
Age, years	62	50–80	Normal	[13]
			(SD =12)	
Male gender, %	77	0–100	Beta	
Proportion of patients with severe MR at baseline in both arms	0.639	0.511–0.766	Beta	
			(α = 23; β = 13)	
Proportion of patients with severe MR between 1 and 5 months in PMVR arm	0.35	0.28–0.42	Beta	
			(α = 12; β = 22)	
Proportion of patients with severe MR from 6 months and onwards in PMVR arm	0.258	0.206–0.309	Beta	
			(α = 8; β = 23)	
Effectiveness data and transition probabilities				
Probability of peri-procedural mortality	0.019	0.01–0.03	Beta	[13]
			(α = 1; β = 52)	
Probability of myocardial infarction, which leads to PCI	0.04	0.02–0.06	Beta	
			(α = 2; β = 51)	
Probability of vessel perforation	0.02	0–0.03	Beta	
			(α = 1; β = 52)	
Probability of unsuccessful percutaneous annuloplasty	0.32	0.05–0.40	Beta	
			(α = 17; β = 36)	
Probability of arrhythmia	0.04	0.02–0.06	Beta	Assumption
			(α = 2; β = 51)	
Six-month probability of excess mortality for NYHA class II	0.04	0.02–0.06	Beta	[22]
			(α = 4; β = 96)	
Six-month probability of excess mortality for NYHA class III	0.07	0.035–0.105	Beta	
			(α = 7; β = 93)	
Six-month probability of excess mortality for NYHA class IV	0.28	0.14–0.42	Beta	
			(α = 28; β = 72)	
Monthly probability of hospitalization for NYHA class I	0.015	0.008–0.023	Beta	[21]
			(α = 1.5; β = 98.5)	
Monthly probability of hospitalization for NYHA class II	0.024	0.012–0.036	Beta	
			(α = 2.4; β = 97.6)	
Monthly probability of hospitalization for NYHA class III	0.024	0.012–0.036	Beta	
			(α = 2.4; β = 97.6)	
Monthly probability of hospitalization for NYHA class IV	0.154	0.077–0.231	Beta	
			(α = 15.4; β = 84.6)	
Relative risk for all-cause mortality with present severe MR	1.5	1.1–1.9	Log-normal (SE_log_ = 0.26)	[23]
Relative risk for hospitalization due to HF with present severe MR	1.7	1.3–2.1	Log-normal (SE_log_ = 0.33)	
Resource utilization and cost data				
Cost of Carillon device, €	18,000	12,600–23,400	-	Cardiac Dimensions, Inc.
Cost of PMVR placement procedure, €	4844	3391–6297	-	G-DRG code F19C
Cost of treatment of vessel perforation after PMVR placement, €	1998	1399–2597	-	
Percentage of patients being hospitalized with stay in intensive care unit	7.2 %	-	Dirichlet	[27]
Percentage of patients being hospitalized with stay in coronary care unit	25.6 %	-		
Percentage of patients being hospitalized with CABG performed	0.3 %	-		
Percentage of patients being hospitalized with PTCA performed	0.2 %	-		
Percentage of patients being hospitalized with heart transplantation performed	2.6 %	-		
Percentage of patients being hospitalized with no procedure performed	62.3 %	-		
Cost of hospitalization with stay in intensive care unit, €	5004	3503–6506	-	G-DRG code F62A 2013
Cost of hospitalization with stay in coronary care unit, €	5004	3503–6506	-	G-DRG code F62A 2013
Cost of hospitalization with CABG performed, €	15,056	10,539–19,573	-	G-DRG code F06E
Cost of hospitalization with PTCA performed, €	3793	2655–4931	-	G-DRG code F56B plus ZE101
Cost of hospitalization with heart transplantation performed, €	86,337	60,436–112,239	-	G-DRG code A05B 2013
Cost of hospitalization with no procedure performed, €	2740	1918–3562	-	G-DRG code F62B 2013
Annual cost of routine management of heart failure in NYHA class I, €	495	258–1031	Gamma	[26]
			(α = 1;λ = 516)	
Annual cost of routine management of heart failure in NYHA class II, €	874	455–1821	Gamma	
			(α = 1;λ = 910)	
Annual cost of routine management of heart failure in NYHA class III, €	864	450–1800	Gamma	
			(α = 1;λ = 900)	
Annual cost of routine management of heart failure in NYHA class IV, €	929	484–1935	Gamma	
			(α = 1;λ = 967)	
Utility data				
Utility for NYHA I class	0.815	0.652–0.978	Beta	[27]
			(α = 395; β = 90)	
Utility for NYHA II class	0.720	0.576–0.864	Beta	
			(α = 662; β = 257)	
Utility for NYHA III class	0.590	0.472–0.708	Beta	
			(α = 360; β = 250)	
Utility for NYHA IV class	0.508	0.406–0.609	Beta (α = 52; β = 50)	
Decrement for percutaneous annuloplasty procedure	0.043	0.034–0.051	Beta	[29]
			(α = 96; β = 2129)	

SD, standard deviation; MR, mitral regurgitation; PMVR, percutaneous mitral valve repair; NYHA, New York Heart Association

The probability of hospitalization due to worsening of heart failure was derived from the literature [21]. Age- and gender-specific general mortality was derived from German life tables [6]. Excessive all-cause mortality in relation with the NYHA class was also derived from the literature [22].

Functional mitral regurgitation is an independent predictor of mortality and hospitalization in patients with chronic heart failure [23–25]. In the model, the additional risk of mortality and hospitalization in patients with severe FMR versus patients with mild/moderate FMR or no FMR was introduced. Functional mitral regurgitation was classified as mild/moderate with an effective regurgitant orifice area (EROA) ≤0.2 cm^2^ and as severe with an EROA ≥0.2 cm^2^.

Patients in the PMVR arm experienced a decrease in FMR and an improvement in NYHA class starting the 1st month after the treatment. The long-term follow-up of these patients (3 years) has demonstrated a stable and durable effect of PMVR over time and, therefore, a sustained FMR reduction was assumed. According to the results of the TITAN trial, the patients in the control group had no improvement in FMR.

An intention-to-treat approach was used to simulate disease progression in the patients with unsuccessful device placement. In this sub-group, the degree of FMR and the NYHA functional classes were similar to the optimal medical treatment arm.

### Resource utilization and cost data

All resource use and cost data were derived from German sources (Table 2). The cost of PMVR was calculated from relevant German diagnosis-related group (DRG) (F19C) with the subtracted cost of implant and the added cost of the Carillon device. Peri-operative myocardial infarction was reported among the 20 most prevalent secondary diagnoses for the DRG, and it was assumed that no additional cost was incurred to manage this event. Based on clinical opinions, 14 additional days of hospital stay would be needed to treat a coronary sinus perforation. The basic DRG tariff covers the treatment for 2 to 10 days of hospital stay (average of 5.2 days). Therefore, incremental costs were applied only for the extra days of hospital stay necessary to treat a vessel perforation. The cost of the device and procedure for the cases of unsuccessful implantation was included in the analysis and was assumed to be covered by the statutory health insurance.

The cost of routine management of heart failure (including the costs of physician, medication and rehabilitation) according to the NYHA class was derived from a recent analysis from the German Competence Network Heart Failure [26]. The costs of hospitalization in the intensive care unit and coronary care unit and the costs of coronary artery bypass grafting, percutaneous transluminal coronary angioplasty, heart transplantation and no invasive procedures were derived from relevant German DRGs. The proportion of hospitalized patients undergoing invasive treatment and the type of invasive treatment were extrapolated from the CARE-HF trial [27].

Only the direct costs were included and were presented in 2013 Euros. The inflation adjustment was performed using the German consumer price index [28].

### Utility data

Utility scores were assigned for each NYHA class irrespective of the treatment received and the hospitalization status. Utility values were derived from the CARE-HF trial [27]. A one-month utility decrement was applied to the Carillon implantation procedure which assumes a similar reduction in the quality of life after percutaneous coronary intervention (0.043) [29].

### Base-case analysis

The analysis was performed from the perspective of the German statutory health insurance over a 10-year time horizon.

The characteristics of the modeled cohort were primarily derived from the TITAN trial: the mean age was 62 years (SD 12 years), 77 % were males, all patients had a NYHA class III (as average NYHA class was 3.0 ± 0.24 in the TITAN trial) and 63 % had severe (MR 4+) FMR.

The incremental cost-effectiveness ratio (ICER) was calculated by comparing the difference in the average total costs and the difference in the average quality-adjusted life years (QALY) among the model’s arms. The intervention was considered cost-effective if the ICER was below the willingness-to-pay threshold of €35,000/QALY [30, 31]. All costs and outcomes beyond the first year were discounted by 3.0 % annually following the recommendations of the German Institute for Quality and Efficiency in Health Care (IQWIG) [32]. The model was constructed in Microsoft Excel 2010 (Microsoft Corp., Redmond, Washington, USA).

### Model validation

After confirming the validity of the results, a number of “stress tests” were performed to evaluate the technical performance of the model. Subsequently, the results of the analysis (all-cause mortality at different follow-ups) were compared with data from the TITAN trial as well as two epidemiological studies of patients with FMR [23, 25].

### Sensitivity analysis

A one-way sensitivity analysis was performed to assess the impact of varying the model parameters while holding other variables fixed at base-case values. The major cost drivers were identified and the results of the sensitivity analysis results were presented by means of a Tornado diagram. In addition, analysis of fade-out effect for PMVR was performed with transition probabilities between NYHA classes in PMVR arm being equal to OMT arm after 3 years from the start of analysis (as maximum length of observation in Carillon studies is 3 years [33]). In addition to the base-case analysis over 10-year time horizon, cost-effectiveness of PMVR was also estimated over a lifetime horizon.

A probabilistic sensitivity analysis was also performed with 5000 Monte Carlo simulations. A beta distribution was used for the probabilities and utility values, a dirichlet distribution was used for the probabilities of transition between NYHA classes and proportions of patients receiving different treatments during hospitalization, and a log-normal distribution was used for the relative risk. DRG tariffs and cost of PMVR were not tested in probabilistic sensitivity analysis, as there was no uncertainty associated with them.

### Ethics statement

Approval by ethics committee was not required, as study did not involve human material or human data.

## Results

### Model validation

**Fig. 2:**
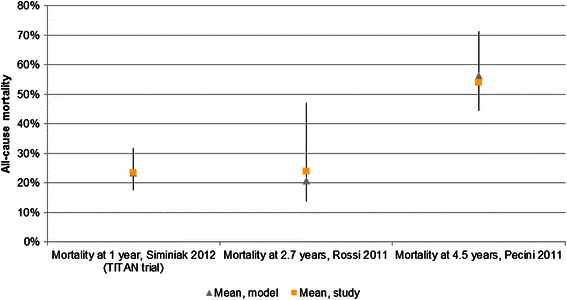
Results of the model validation. Scares represents the mean value from the validation study, triangles represent the mean value from the model, and lines represent the 95 % credible interval for the modeled results

Validation of the model showed that the model could precisely predict all-cause mortality at one, two and five years of follow-up (Fig. 2).

### Base-case analysis

Over 10 years, the total cost in the PMVR arm was €36,785 and €18,944 in the optimal medical treatment arm (Table 3). Percutaneous mitral valve repair, however, provided a significant benefit to the patients compared with the optimal medical treatment (1.15 incremental QALYs and 1.41 incremental life years). Cumulative mortality was 13.8 % and 23.5 % at 1 year, 20.0 % and 33.0 % at 2 years, 35.6 % and 54.3 % at 5 years, and 55.5 % and 76.5 % at 10 years in the PMVR and optimal medical treatment arms respectively.

**Table 3:** Results of the cost-utility analysis

Treatment arm	Cost, €	Incremental cost, €	LYG	Incremental LYG	QALY gained	Incremental QALY gained	ICER, €/QALY
Optimal medical treatment	18,944		4.46		2.91		
Percutaneous mitral valve repair	36,785	17,841	5.87	1.41	4.06	1.15	15,533

ICER – incremental cost-effectiveness ratio; LYG – life years gained; QALY – quality-adjusted life years

The percutaneous mitral valve repair treatment was cost-effective in comparison to optimal medical treatment with an ICER of €15,533/QALY.

### Sensitivity analysis

**Fig. 3:**
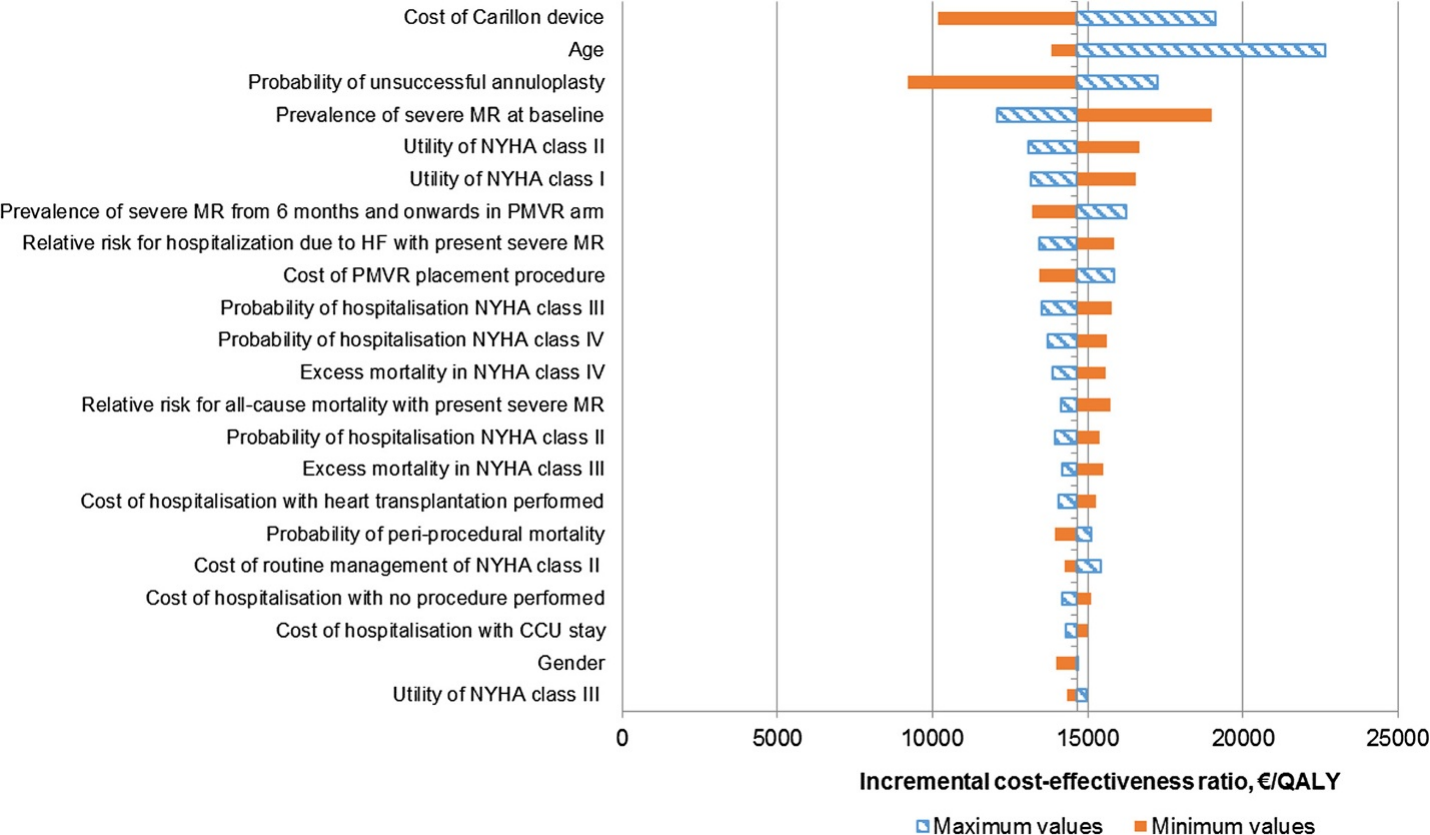
Tornado diagram. The Tornado diagram shows the results of the sensitivity analyses on the cost-effectiveness of PMVR compared with optimal medical treatment. The blue lines with a diagonal pattern reflect results while using maximum values in the sensitivity analysis and the orange lines reflect results while using minimum values. The vertical line indicates the base-case incremental cost-effectiveness ratio. PMVR, percutaneous mitral valve repair; QALY, quality-adjusted life year

The results of the one-way sensitivity analysis were stable with no single variable altering the cost-effectiveness of PMVR (Fig. 3). The most sensitive variables were the cost of the Carillon device, the age of the patient, the probability of unsuccessful annuloplasty and the presence of severe FMR at baseline.

In the analysis over lifetime horizon PMVR led to incremental cost of €19,539, incremental life-years gained of 3.23 and QALYs gained of 2.47. PMVR was more cost-effective over lifetime (ICER of €7,914/QALY) compared with base-case analysis over 10-year time horizon.

Analysis of fade-out effect for effectiveness of PMVR showed that technology remains cost-effective with ICER of €23,582/QALY. In this analysis, PMVR led to an incremental cost of €20,662 (cost of €39,702 and €19,040 in the PMVR and optimal medical management arms respectively), an incremental life years gained of 1.04 (life years gained of 5.49 and 4.46 in the PMVR and optimal medical management arms respectively) and an incremental QALYs gained of 0.88 (QALYs gained of 3.78 and 2.90 in the PMVR and optimal medical management arms respectively).

**Fig. 4:**
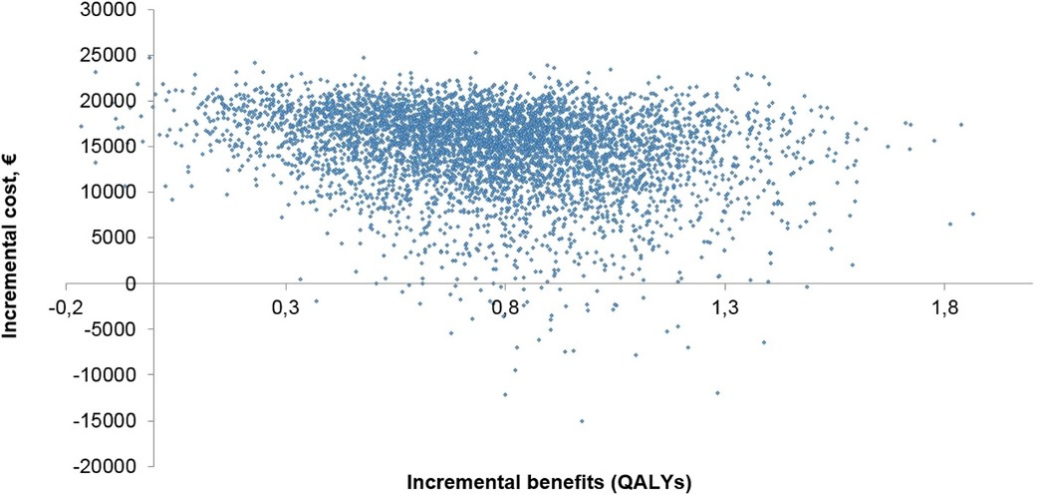
Cost-effectiveness acceptability plane. QALY, quality-adjusted life year

**Fig. 5:**
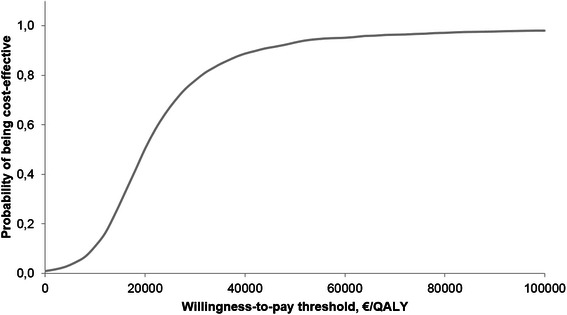
Cost-effectiveness acceptability curve. QALY, quality-adjusted life year

In the probabilistic sensitivity analysis with 5000 simulations, PMVR led to an incremental cost of €14,868, an incremental life-years gained of 0.81 and a QALYs gained of 0.77 (Fig. 4). Percutaneous mitral valve repair was cost-effective compared with optimal medical treatment with an ICER of €19,414/QALY. At a willingness-to-pay threshold of €35,000/QALY, PMVR had a 84 % probability of being cost-effective (Fig. 5). At a threshold of €50,000/QALY, PMVR had a 93 % probability of being cost-effective.

## Discussion

Patients with left ventricular dysfunction and FMR have a poor prognosis [2, 3, 24, 25, 34]. In severely ill patients, the number of treatment options is limited. A survey of European patients with heart failure and mitral regurgitation revealed that the determinants of preclusion of surgical treatment include impaired LVEF, older age and comorbidities [35], which are typical for heart failure patients with FMR.

Furthermore, patients with FMR have a normal mitral apparatus because the pathophysiological mechanism leading to mitral regurgitation is left ventricular dilatation with the subsequent mal-coaptation of the mitral leaflets. This has resulted in the ongoing evaluation of an array of non-surgical methods to attempt to correct mal-coaptation [5, 36].

Our study showed that PMVR using Carillon® Mitral Contour System® leads to an incremental cost (€17,841), but it also leads to significant additional clinical benefit to patients with increments of QALY and life-years of 1.15 and 1.41, respectively. Overall, PMVR can be cost-effective in German settings with an ICER of €15,533/QALY.

The model utilized a Markov process on the basis of four NYHA functional classes with each class sub-divided into hospitalized and non-hospitalized states. Many published decision analytic models in the field of heart failure have utilized NYHA classes to trace the progression of symptoms [22, 27, 37–39]. Hospitalizations are also a common determinant in economic evaluations, as they are one of the main cost drivers of heart failure. Studies have shown that hospitalizations account for 60–74 % of the total expenditures of heart failure in France, the UK, The Netherlands, New Zealand and Sweden [40–42]. A recent analysis from Germany reports that the annual cost of heart failure in Germany totals €2.8 billion of which €1.7 billion is spent on in-patient facilities [43].

Results of the study are similar to published economic evaluations of another percutaneous treatment for mitral regurgitation, the MitraClip (Abbott Vascular, Santa Clara, California) [44, 45]. The analyses, based on the Endovascular Valve Edge-to-Edge REpair High Risk Study [46], were performed in the UK [44] and Canadian [45] settings and both compared the MitraClip to optimized medical treatment. In the UK study, over 10-year time horizon, the MitraClip lead to an incremental QALYs of 2.04 and a resulting ICER of £14,800/QALY. The Canadian study employed a lifetime horizon for its base-case analysis and reported an incremental QALYs of 1.73 and a resulting ICER of $23,433/QALY. At a 10-year time horizon, the MitraClip was also cost-effective with an ICER of $25,752/QALY. In our study, a similar level of clinical benefits (incremental QALYs of 1.15) and cost-effectiveness (ICER of €15,533/QALY) were demonstrated.

Our study has several limitations. First, the transition probabilities between the different NYHA functional classes were based on a limited sample from the TITAN trial. Due to the limited sample size, not all transitions were possible in the base-case analysis. This limitation was addressed in the probabilistic sensitivity analysis. Second, the model utilized clinical and surrogate (mitral regurgitation) outcomes to predict mortality and hospitalization. Ideally, data on the impact of PMVR on mortality and hospitalizations as well as the utility data should be obtained directly from prospective randomized controlled trials rather than extrapolated from multiple sources. Evidence in the field of FMR is increasing and a more precise assessment may be possible in the future. A third limitation relates to the nature of the comparative effectiveness data for PMVR. Data about change of level of mitral regurgitation were obtained from the TITAN trial [13], in which the control group consisted of patients who underwent unsuccessful device placement, with explant of Carillon occurring during the procedure. Ideally, the effectiveness data should be obtained from a randomized controlled trial with an intention-to-treat analysis to allow the assessment of outcomes despite the real treatment received in groups with balanced observed and unobserved baseline characteristics. Nevertheless, in TITAN trial both groups had similar baseline demographic and clinical characteristics. However, due to the absence of random allocation into the treatment groups, there might be unobserved differences between groups, which may impact the reported effectiveness of PMVR. The TITAN trial had a sufficient follow-up period and the differences in the studied FMR measures at 1, 6 and 12 months between groups are not likely explained by reasons other than the impact of the implanted device. Finally, the maximum length of observation in Carillon studies is 3 years [28] while the clinical effectiveness was extrapolated over a 10-year time horizon in our model. Nevertheless, analysis of fade-out effect for PMVR with effectiveness limited to 3 years showed that technology remains cost-effective in comparison with optimal medical treatment (ICER of €23,582/QALY). The aforementioned limitations should be taken into consideration when results of the present model are used to inform clinical or payer decision-makers.

## Conclusions

When compared with optimal medical treatment, PMVR using the Carillon® Mitral Contour System® may be cost-effective in inoperable patients with congestive heart failure who have moderate to severe FMR.
